# A Comprehensive Review on the Phytochemical Constituents and Pharmacological Activities of *Pogostemon cablin* Benth.: An Aromatic Medicinal Plant of Industrial Importance

**DOI:** 10.3390/molecules20058521

**Published:** 2015-05-12

**Authors:** Mallappa Kumara Swamy, Uma Rani Sinniah

**Affiliations:** Department of Crop Science, Faculty of Agriculture, Universiti Putra Malaysia, Serdang, Selangor, Darul Ehsan 43400, Malaysia; E-Mail: swamy.bio@gmail.com

**Keywords:** *Pogostemon cablin*, biological activities, phytomedicine, essential oil

## Abstract

*Pogostemon cablin* Benth. (patchouli) is an important herb which possesses many therapeutic properties and is widely used in the fragrance industries. In traditional medicinal practices, it is used to treat colds, headaches, fever, nausea, vomiting, diarrhea, abdominal pain, insect and snake bites. In aromatherapy, patchouli oil is used to relieve depression, stress, calm nerves, control appetite and to improve sexual interest. Till now more than 140 compounds, including terpenoids, phytosterols, flavonoids, organic acids, lignins, alkaloids, glycosides, alcohols, aldehydes have been isolated and identified from patchouli. The main phytochemical compounds are patchouli alcohol, α-patchoulene, β-patchoulene, α-bulnesene, seychellene, norpatchoulenol, pogostone, eugenol and pogostol. Modern studies have revealed several biological activities such as antioxidant, analgesic, anti-inflammatory, antiplatelet, antithrombotic, aphrodisiac, antidepressant, antimutagenic, antiemetic, fibrinolytic and cytotoxic activities. However, some of the traditional uses need to be verified and may require standardizing and authenticating the bioactivity of purified compounds through scientific methods. The aim of the present review is to provide comprehensive knowledge on the phytochemistry and pharmacological activities of essential oil and different plant extracts of patchouli based on the available scientific literature. This information will provide a potential guide in exploring the use of main active compounds of patchouli in various medical fields.

## 1. Introduction

A major segment of the flora includes medicinal and aromatic plants which are the source of raw materials used in the pharmaceutical, fragrance, cosmetic, flavor and perfumery industries. In spite of much progress made in synthetic drug research, plants and their products are still considered to be the major sources of medicaments and have extensive use in the pharma industry [1,2]. Most modern medicines are derived from plants and their products obtained by applying modern technologies to traditional practices [3]. The use of plants is customary in Indian systems of medicine like Ayurveda, Unani, Sidda and many other indigenous and folk practices [4]. *Pogostemon cablin* Benth. (patchouli), belonging to the family Lamiaceae is an aromatic herb having immense commercial importance. Patchouli plants have been widely used in traditional medicinal practices in India and China to treat many medical ailments. In Chinese medicine, it has been used to remove dampness, relieve summer heat and exterior syndrome, and as an anti-emetic and appetite stimulant [5]. Patchouli plant is a component of the traditional Chinese medicine, Pogostemoni Herba which is widely used in the cosmetic and hygiene industries. Chinese traditional formulae, such as Baoji Pill and Houdan Pill containing patchouli herb are used in treating inflammatory diseases [6,7]. The plant is included in the preparations of Indian Ayurvedic treatments such as Rasa, Guna and Virya. In China, Japan and Malaysia, it is used to treat colds, headaches, nausea, vomiting, diarrhea, abdominal pain, insect and snake bites [5]. The commercial importance of patchouli is due to its oil, which can be obtained by steam distillation of the shade dried leaves. It possesses a powerful sweet, herbaceous aromatic, spicy fragrance. [8]. Among all the essential oil yielding plants, patchouli is considered to have tremendous business potential in the global market because of its unique flavor and fragrance properties, and also biological activities [9,10,11]. Though it is not a dominant source of fragrance, its ability to blend with other essential oils gives a strong base, lasting character, fixative properties and aids in preventing evaporation thus promoting tenacity. Hence, the oil is widely used in the manufacturing of soaps, scents, body lotions and detergents [12]. Patchouli oil is used in aromatherapy to relieve depression, stress, calm nerves, control appetite and to improve sexual interest. Patchouli also possesses insecticidal, antibacterial and antifungal properties [13,14,15]. Some of its other biological activities include antimicrobial, antioxidant, analgesic, anti-inflammatory, antiplatelet, antithrombotic, aphrodisiac, antidepressant, antimutagenic, antiemetic, fibrinolytic and cytotoxic activities [13,16,17]. Therefore, in this review, a compilation of literature is made in order to produce a comprehensive report related to phytochemistry of different plant parts of *P. cablin* and various biological properties exhibited by purified compounds as well as by the crude extracts. An updated research survey was carried out by using various search engines like Google Scholar, Scopus, PubMed and ScienceDirect. 

## 2. Botanical Description

Patchouli is a native of the Philippines and grows wild in many South Asian countries, and is presently cultivated on a commercial scale in India, Indonesia, Malaysia, China, Singapore, West Africa and Vietnam [18,19]. The word ‘cablin’ is derived from ‘cabalam’ which is also a local name for the patchouli plant in the Philippines and these are synonymous [20]. About 25 species of *Pogostemon* are reported to occur in India. Patchouli is also known as patchouly, *tamala patra* in Sanskrit, *patcholi* in Hindi, *patche tene* in Kannada, *pacchilai* in Tamil, *patchilla* in Malayalam, *patchapan* or *patcha* in Marathi and *guang hou xiang* in Chinese, *nilam* in Malaysia and Indonesia, *phimsen* in Thailand. Morphologically, patchouli is a hardy perennial herb adapted to hot and humid climatic conditions. It grows up to 1 to 1.2 m height with an erect stem and broad leaves (0.85 inches). The margins of the leaves are lobed and abundant hairs are present on its dorsal surface [21]. The leaves are known to accumulate essential oil in the glandular trichomes [22]. The plant bears small pale pink-white flowers [13].

## 3. Phytochemistry 

The potential benefits of *P. cablin* have been well explored in recent years by many scientific communities. Various studies on its chemistry and biological activities are well documented and more recent research studies are focused on isolating individual compounds to understand their mechanism involved in various pharmacological activities. The title plant contains various phytochemicals including many monoterpenoids, triterpenoids, sesquiterpenoids, phytosterols, flavonoids, organic acids, lignins, glycosides, alcohols and aldehydes. 

### 3.1. Volatile Chemical Composition of P. cablin

**Figure 1:**
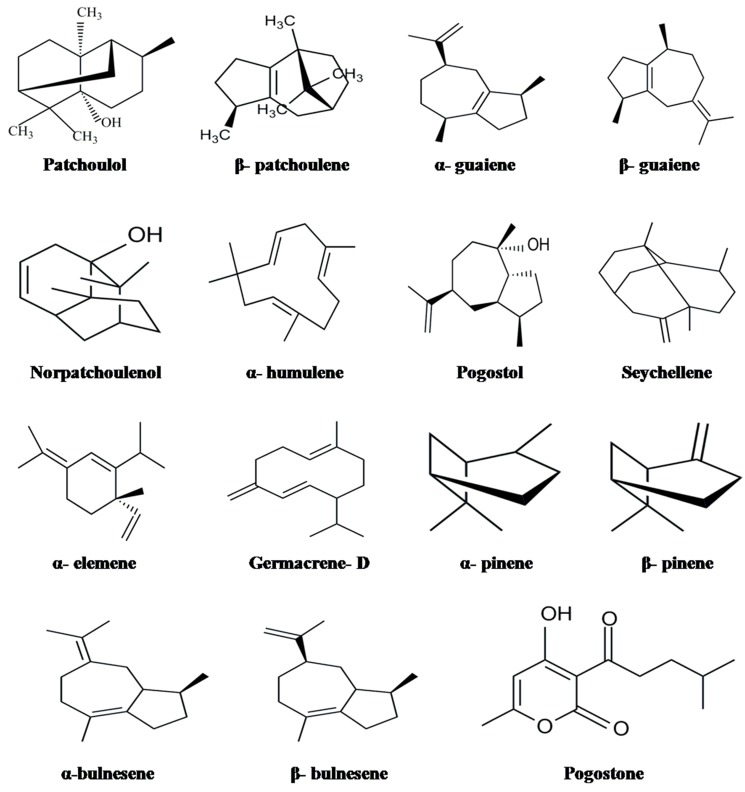
The structures of some of the volatile chemical constituents.

A major constituent obtained from the leaves of *P. cablin* is the essential oil (patchouli oil) which possesses many pharmacological properties. The curative action of patchouli oil is directly related to its qualitative as well as quantitative chemical constituents. The known volatile chemical components of patchouli oil are presented in Table 1, while the structures of some major and important compounds are depicted in Figure 1. Generally, steam or fractional distillation is used to obtain patchouli oil [23,24], although these processes cause the thermal degradation of a few of the chemical constituents present in the oil. Therefore, Donelian *et al.* [25] established the supercritical carbon dioxide extraction method to obtain the maximum yield of patchouli oil with better extraction quality. Molecular distillation has also been reported by Hu *et al.* and Chen *et al.* [26,27]. However, microwave assisted extraction using ethanol as solvent improves oil extraction compared to other conventional methods as it employs a precise computer controlled high temperature in a closed vessel equipped with a magnetic stirrer [28]. 

Patchouli oil is rich in sesquiterpenes, mainly the patchouli alcohol (patchoulol), a tricyclic sesquiterpene which is widely used in perfumery products, soaps and other cosmetic goods [29]. Patchoulenes, guaiene, seychellene are few other sesquiterpene hydrocarbons also characterize the aroma of patchouli oil. According to many researchers, patchoulol and α-patchoulene are the major constituents which regulate and control patchouli oil quality [25,29,30]. Several other minor sesquiterpenes, caryophyllene, pogostol, α-, β-, γ- and δ-patchoulene, seychellene, cycloseychellene, α- and β-bulnesene, α- and β-guaiene and norpatchoulenol are also reported in patchouli oil [31,32]. The biological activities of patchouli oil are strongly associated with the chemical constituents such as pogostone, patchoulol, α- and β-patchoulene. Previous studies have stated that pogostone as one of the major chemical constituent of patchouli oil largely responsible for the intense aromatic odor and more recently this compound has been demonstrated to exert many pharmaceutical activities [33,34,35]. Later on, the chemical structure of pogostone present in the patchouli oil was revealed by Zheng *et al.* [36]. 

**Table 1:** The volatile constituents of *P. cablin*.

Compound Name	Formula	Analytical Method	References
Aciphyllene *	C_15_H_24_	GCMS	[37,38]
Alloaromadendrene	C_15_H_24_	GC	[39]
Aromadendrene	C_15_H_24_	GC	[39]
β-Bourbonene	C_15_H_24_	GC×GC–TOF MS	[40]
α- and β-Bulnesene	C_15_H_24_	GCMS;NMR	[31,32,41,42,43,44,45,46]
(+)-Camphene	C_10_H_16_	GC	[39]
(−)-Camphor	C_10_H_16_O	GC×GC–TOF MS	[40]
δ-Cardinene	C_15_H_24_	GC;GCMS;NMR	[40,41]
α-Caryophyllene (α-Humulene)	C_15_H_24_	GCMS;NMR	[11,31,32,37,38,39,40,41,42,44,45,47]
β-Caryophyllene *	C_15_H_24_	GCMS	[37,48]
*trans*-Caryophyllene *	C_15_H_24_	GCMS	[37,44,49]
Caryophyllene oxide	C_15_H_24_O	GC×GC–TOF MS;GCMS	[36,38,40]
Copaene	C_15_H_24_	GC	[11,38]
β-Cubebene	C_15_H_24_	GCMS	[45,46]
β-Copaen-4-α-ol	C_15_H_24_O	GCMS	[44]
Cycloseychellene	C_15_H_24_	GC;GCMS	[31,32,38]
Diidro-aromadendrane	C_15_H_26_	GCMS	[44]
α-, β- and δ-Elemene	C_15_H_24_	GC;GCMS; NMR	[11,38,39,40,41,44,45]
Elemol	C_15_H_26_O	GC×GC–TOF MS	[40]
Epiglobulol	C_15_H_26_O	GC×GC–TOF MS	[40]
Eucalyptol	C_10_H_18_O	GC×GC–TOF MS	[40]
α-Elemenone	C_15_H_22_O	GC×GC–TOF MS	[40]
Epifriedelinol	C_30_H_52_O	NMR;IR;MS;UV	[50]
7-Epi-α-selinene	C_15_H_24_	GCMS	[40,47]
*cis*-Farnesol	C_15_H_24_O	GC×GC–TOF MS	[40]
Friedelin	C_30_H_50_O	NMR;IR;MS;UV	[11]
Germacrene- A, B, D	C_15_H_24_	GC/GCMS	[11,38,45,46]
Globulol	C_15_H_26_O	GC×GC–TOF MS	[40,46]
α-, β- and δ-Guaiene	C_15_H_24_	GC;GCMS; NMR	[11,31,32,36,37,38,39,40,41,42,45,47]
α-, γ-Gurjunene	C_15_H_24_	GC; GCMS	[38,50]
Heptanal	C_7_H_14_O	GC×GC–TOF MS	[40]
Limonene	C_10_H_16_	GC;GCMS; GC×GC–TOF MS	[38,39,40]
Longipinanol	C_15_H_26_O	GCMS	[47]
Myrtenol	C_10_H_16_O	GC×GC–TOF MS	[40]
Nonanal	C_9_H_18_O	GC×GC–TOF MS	[40]
Norpatchoulenol	C_14_H_22_O	GCMS	[11,31,32,40]
1-Octen-3-ol	C_8_H_16_O	GCMS	[43,47]
3-Octanol	C_8_H_18_O	GC×GC–TOF MS	[36,39]
Oleanolic acid	C_30_H_48_O_3_	NMR;IR;MS;UV	[39]
Patchouli alcohol **	C_15_H_26_O	GC;GCMS; NMR	[11,31,32,38,39,40,41,43,44,46,47]
α-,β-,γ- and δ-Patchoulene	C_15_H_24_	GCMS; GC;GCMS; NMR	[31,32,36,37,39,40,41,42,43,44,46,47,50]
β-Phellandrene	C_10_H_16_	GC×GC–TOF MS	[40]
α- and β-Pinene	C_10_H_16_	GC;GCMS	[36,39,40,47,50]
Pogostol	C_15_H_26_O	GC;GCMS; NMR	[31,32,39,40,41,50]
Pogostone	C_12_H_16_O_4_	NMR;IR;MS;UV	[11,36,40,41,43,44,47,49,50,51]
α- and β-Selinene	C_15_H_24_	GC×GC–TOF MS	[36,40,45,46,47]
Seychellene	C_15_H_24_	GC;GCMS; NMR	[31,32,37,39,40,41,42,44,46,47,50]
Spathulenol	C_15_H_24_O	GCMS;GC×GC–TOF MS	[40,44]
(−)-α-Terpineol	C_10_H_18_O	GC×GC–TOF MS	[40]
Valencene	C_15_H_24_	GCMS	[47]
Viridiflorene	C_15_H_26_O	GCMS	[47]

Note: * and ** represents compounds obtained from stem/leaf/oil and whole herb/stem/leaf/oil respectively. Unmarked compounds are obtained from leaf/oil.

The chemical composition of patchouli oil varies among samples collected from different geographic locations. Li *et al.* [52] revealed the significant effect of different habitats, collection periods, and processing methods on the volatile oil yield and its main constituents. Similarly, the oil yield is also influenced by different collection times. It is reported that contents of volatile oil obtained from leaves harvested from June to August and cultivated in Hainan, China were 0.8%, 0.7% and 0.6%, respectively, while the patchouli alcohol content was highest in the month of June [53]. Gas chromatography (GC), Gas chromatography/mass spectroscopy (GC/MS) and nuclear magnetic resonance (NMR) were used for studying the chemical composition of patchouli oil from Vietnam. The major compounds identified were α-, β- and δ-patchoulene, β-elemene, β-caryophyllene, α- and δ-guaiene, seychellene, α-bulnesene, δ-cardinene, pogostol and patchouli alcohol. The presence of 32%–37% patchouli alcohol content was found to be more odor intensive component of the essential oil [41]. However, in the Philippines, it was predicted that a distinct aroma is due to the occurrence of germacrene-B, a new sesquiterpene identified as the major component of the patchouli oil [11]. Later, Rakotonirainy *et al.* [42] used 1- and 2-dimensional NMR and GC/MS to derive the structural formulae of the compounds, including α-patchoulene, α-bulnesene, α-guaiene, and seychellene.

The essential oil from *P. cablin* plants collected from China (Gaoyao County, Guangdong Province) and its volatile chemical compositions were analyzed by GC/MS. The study revealed the presence of pogostone (30.99% in stems, 21.31% in leaves), patchouli alcohol (10.26% in stems, 37.53% in leaves), *trans*-caryophyllene (4.92% in stems, 6.75% in leaves), α-guaiene (2.27% in stems, 6.18% in leaves) and seychellene (1.56% in stems, 1.99% in leaves) as the main constituents [49]. Similarly, GC/MS analysis of essential oil extracted from both leaves and stems of Patchouli plants collected from the Leizhou County of China revealed sesquiterpenes such as patchouli alcohol, α-guaiene, δ-guaiene, α-patchoulene, seychellene, aciphyllene and *trans*-caryophyllene [37]. Guan *et al.* [50] have identified nine sesquiterpene compounds, namely patchouli alcohol, pogostone, frieddelin, epifriedelinol, pachypodol, retusine, oleanolic acid, β-sitosterol and daucosterol on the basis of spectral data. In the same year, Kang *et al.*, developed an efficient method to isolate and determine the patchoulol constituent quantitatively from *P. cablin* [39]. 

Patchouli oil analyzed by comprehensive two–dimensional gas chromatography time-of-flight mass spectrometry contained mainly monoterpenes and sesquiterpenes [40]. The identified compounds were; (−)-β-pinene, β-pinene, β-phellandrene, δ-elemene, limonene, eucalyptol, 3-octanol, heptanal, nonanal, (−)-camphor, β-bourbonene, β-elemene, α-guaiene, 4-terpinenol, (−)-α-terpineol, α-patchoulene, β-patchoulene, α-caryophyllene, δ-guaiene, β-selinene, δ-cadinene, myrtenol, caryophyllene oxide, elemol, α-elemenone, globulol, epiglobulol, patchoulol, (−)-spathulenol, ledene oxide-(I), *cis*-farnesol and pogostone. A GC and GC/MS study of Indonesian patchouli oil indicated the presence of the following compounds; α-pinene, δ-patchoulene, β-pinene, aciphyllene, limonene, δ-guaiene, δ-elemene, 7-*epi*-α-selinene, α-copaene, norpatchoulenol, α-patchoulene, 1,10-epoxy-11-bulnesene, β-elemene, caryophyllene oxide, cycloseychellene, nortetrapatchoulol, β-caryophyllene, patchouli alcohol, α-guaiene, patchoulenone, seychellene, 9-oxopatchoulol, α-humulene, pogostol, α-patchoulene, isopatchoulenone, γ-gurjunene, and germacrene D [38]. A successful GC/MS method for determining patchoulol content in the dried samples of patchouli was developed and demonstrated by Zhao *et al.* [54]. 

Patchouli plants collected from different cultivation regions and harvested at different times showed differences in their volatile oil compositions [43,44,47]. Thus, *P. cablin* is differentiated into 2-chemotypes namely, pogostone type and patchouliol type on the basis of chemical differences in their volatile oil composition [43]. The GC/MS profile of the patchouli oils collected at different time periods during 24 h showed the presence of 1-octen-3-ol, β-patchoulene, β-elemene, *trans*-caryophyllene, α-guaiene, γ-patchoulene, α-hmulene, α-patchoulene, dihydroaromadendrane, *trans*-β-guaiene, α-bulnesene, β-copaen-4-α-ol, and patchouli alcohol. However, they did not present any significant difference as a function of the harvest time [44]. The chemical profile of patchouli oil collected from different regions of China was identified with nine compounds, namely α-guaiene, β-guaiene, δ-guaiene, β-patchoulene, caryophyllene, seychellene, spathulenol, patchouliol and pogostone. Based on the GC profiles, 18 samples were categorized into three major groups, patchouliol type, pogostone type and interim type [47]. Among the various compounds identified from patchouli oil using GC/MS, patchoulol, germacrene-A, α-guaiene, α-bulnesene, β-patchoulene and patchouli alcohol were the major components while β-pinene, β-elemene, β-caryophyllene, α-patchoulene, β-cubebene and α-selinene were the minor components [45,46]. 

Zeng *et al.* [36] studied and compared the volatile chemical components of *P. cablin* from different culture varieties to produce a chromatographic fingerprint and identified the structure of pogostone. They isolated eight new compounds, including *trans*-famisol, 1-(propen-2-yl)-4-methylspiro-(4,5)-decan-7-one, 1,2,3,4-tetrahydro-1,6-dimethyl-4-(1-methyl)ethylnapthalene, 1,2,3,4,4a,5,6,8a-octa- hydro-4a,8-dimethyl-2-(1-methylethenyl)-2*R*-(2α,4a,α,8a,β)-napthalene, 1,8,8,10-tetramethylcyclo-decene, aristolone, 2,4,5-trimethoxy-1-propenylbenzene and 1,2,4,6-tetramethyltricyclo[5,4,0,0(3,9) undecan-2-antiol. The essential oil of *P. cablin* contained 11 compounds while essential oils of *P. travancoricus* contained 13 compounds. The components such as α-patchoulene and β-patchoulene, patchoulol, β-caryophyllene, α-guaiene, seychellene and selinene were common to both species. However, *P. travancoricus* had a relatively lesser quantity [48]. It was found that lab-scale produced oil had a maximum patchoulol content compared to other commercial patchouli samples [55]. More recently, Li *et al.* [51] developed a more sensitive, precise and accurate HPLC-DAD method to quantify the volatile compound, pogostone and eight other non-volatile flavonoids such as apigenin, rhamnetin, ombuine, 5-hydroxy-7,3ʹ,4ʹ-trimethoxyflavanone, 4ʹ,5-dihydroxy-3,3ʹ,7-trimethoxyflavone, 3,5-dihydroxy-7,4ʹ-dimethoxyflavone, 5-hydroxy-3,3ʹ,4ʹ,7-tetramethoxyflavone and 5-hydroxy-3,4ʹ,7-trimethoxyflavone. 

### 3.2. Non-Volatile Chemical Composition of P. cablin

**Figure 2:**
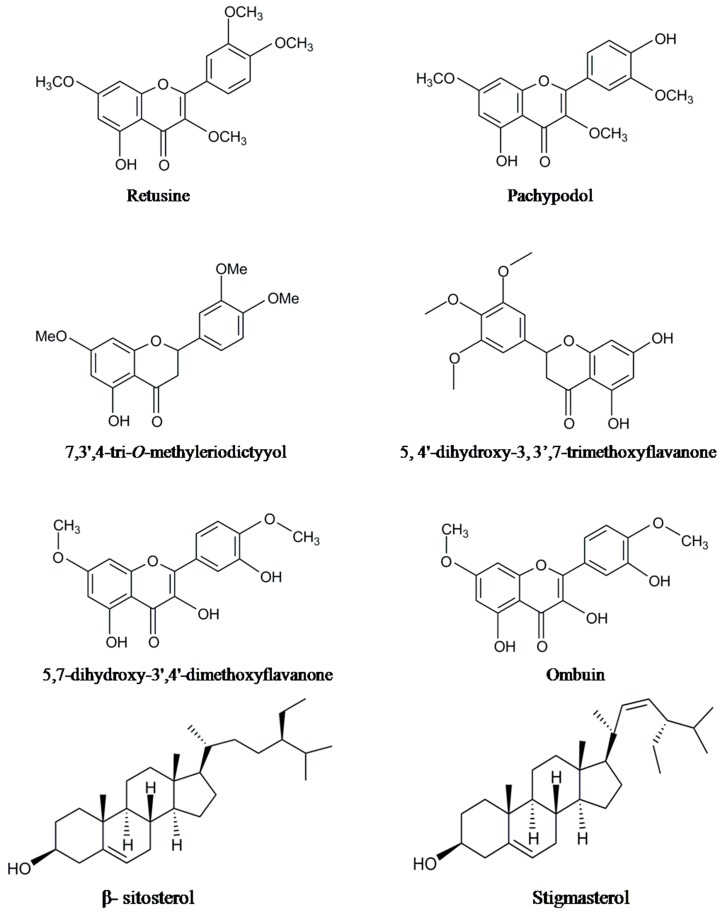

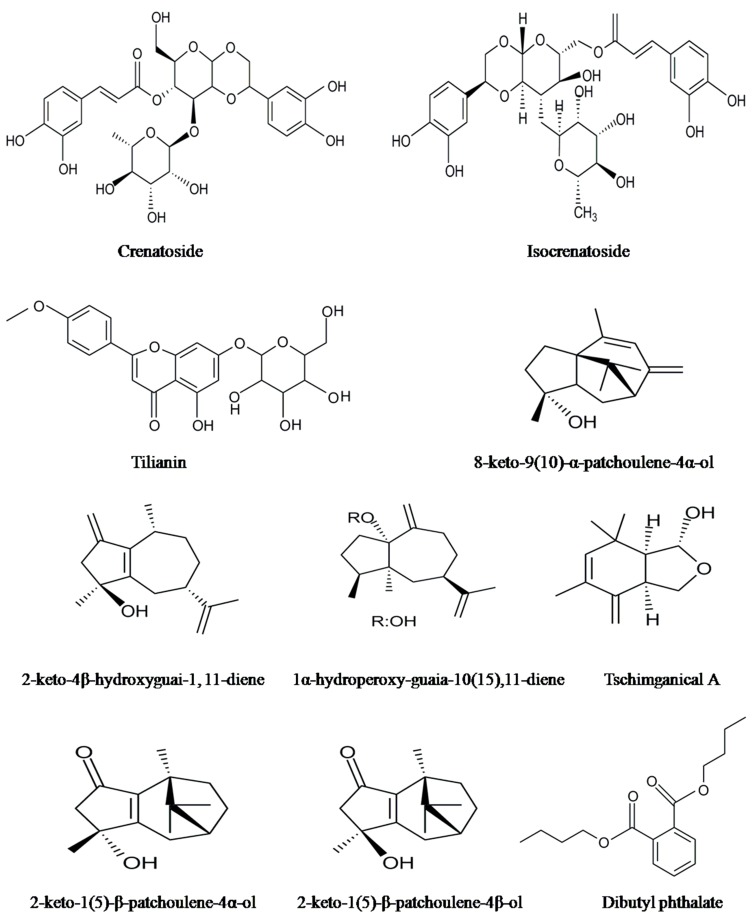
The structures of some of the non-volatile chemical constituents.

Various phytochemicals belonging to flavonoids, glycosides, triterpenes, sesquiterpenes, lignins, aldehydes, and organic acids along with few other constituents were found as major and minor non-volatile components of *P. cablin* (Table 2). The structures of some major and important compounds are illustrated in the Figure 2. Park *et al.* [56] isolated and identified three flavonoids by using cytotoxicity-guided fractionation and spectral analysis. The identified non-volatile compounds were licochalcone A, ombuin and 5,7-dihydroxy-3ʹ,4ʹ-dimethoxyflavanone. Using a TLC and HPLC technique, Amakura *et al.* [57] assessed the quality of Pogostemoni Herba, a crude drug used in Chinese Kampo medicine and developed a potential chemical marker from methanol extracts of dried leaves of *P. cablin.* They isolated the first time three phenylethanoids (isoacteoside, acteoside and crenatoside) from this plant. Using a column chromatography technique and spectral data, nine non-volatile chemical compounds were isolated from methanol leaf extracts of patchouli and identified as epifriedelinol, 5-hydroxymethyl-2-furfural, succinic acid, β-sitosterol, daucosterol, 3ʹʹʹ-*O*-methyl-crenatoside, crenatoside, isocrenatoside, and apigenin-7-*O*-β-d-(6ʺ-*p*-coumaryl)-glucoside [58]. Among these compounds, 5-hydroxymethyl-2-furfural, succinic acid, crenatoside, 3ʹʹʹ-*O*-methylcrenatoside and isocrenatoside were identified for the first time in the genus Pogostemon. Five target compounds, including pogostone and four flavonoids 4ʹ,5-dihydroxy-3ʹ,7-dimethoxyflavanone, 5,4ʹ-hihydroxy-3,7,3ʹ-trimethoxy-flavanone, 5-hydroxy-7,3ʹ,4ʹ-trimethoxyflavanone, 5-hydroxy-3,7,3ʹ,4ʹ-tetrmethoxyflavanone were obtained from the ethanol and hexane extracts of *P. cablin* by using high speed countercurrent chromatography and preparative HPLC techniques [59]. 

**Table 2:** The non-volatile constituents of *P. cablin.*

Compound Name	Formula	Plant Part	Analytical Method	References
**Flavonoids**				
Acacetin	C_16_H_12_O_5_	Stem (Ethanol extract)	IR; HR; NMR	[60]
Apigenin	C_15_H_10_O_5_	Air dried root (Ethanol extract)	HPLC-DAD	[51,58,60]
Diosmetin-7-*O*-β-d-gluco-pyranoside	C_22_H_22_O_11_	Stem (Ethanol extract)	IR; HR; NMR	[60]
4ʹ,5-Dihydroxy-3,3ʹ,7-trimethoxyflavone	C_18_H_16_O_7_	Stem/Root (Ethanol extract)	IR; HR; NMR; HPLC-DAD	[51,60]
4ʹ,5-Dihydroxy-3ʹ,7-dimethoxyflavanone	C_17_H_16_O_6_	Whole plant (Ethanol & hexane extracts)	HSCCC; prep-HPLC;NMR	[59]
5,4ʹ-Dihydroxy-3,3ʹ,7-trimethoxyflavanone	C_18_H_18_O_7_	Whole plant/Root (Ethanol & hexane extracts)	HSCCC; prep-HPLC; NMR; HPLC-DAD	[51,59]
3,5-Dihydroxy-7,4ʹ-dimethoxyflavanone	C_29_H_36_O_15_	Whole plant/Root (Ethanol & hexane extracts)	HSCCC; prep-HPLC; NMR; HPLC-DAD	[51]
5,7-Dihydroxy-3ʹ,4ʹ-dimethoxyflavanone	C_29_H_36_O_15_	Aerial parts (Methanol extract)	UV; IR; MS; NMR	[56]
5-Hydroxy-3,3ʹ,4ʹ,7-tetramethoxyflavone	C_19_H_18_O_7_	Stem (Ethanol extract)	IR; HR; NMR	[51,60]
5-Hydroxy-7,3ʹ,4ʹ-trimethoxyflavanone	C_19_H_20_O_7_	Whole plant/Root (Ethanol & hexane extracts)	HSCCC; prep-HPLC; NMR; HPLC-DAD	[51,59]
5-Hydroxy-3,7,3ʹ,4ʹ-tetrmethoxyflavanone	C_19_H_18_O_6_	Whole plant/Root (Ethanol & hexane extracts)	HSCCC; prep-HPLC; NMR; HPLC-DAD	[51,59]
Licochalcone A	C_21_H_22_0_4_	Aerial parts (Methanol extract)	UV; IR; MS; NMR	[56]
Ombuin	C_17_H_14_O_7_	Air dried aerial parts/Root (Ethanol extract)	HPLC-DAD; IR; MS; NMR	[51,56]
Rhamnetin	C_16_H_12_O_7_	Air dried root(Ethanol extract)	HPLC-DAD	[51]
Retusine	C_19_H_18_O_7_	Leaves	NMR; IR; MS; UV	[61]
**Phytosterols**				
5α-Stigmast-3,6-dione	C_29_H_48_O_2_	Aerial parts	Spectroscopy	[61]
Daucosterol	C_35_H_60_O_6_	Leaves	NMR; IR; MS; UV	[58]
β-Sitosterol	C_29_H_50_O	Dried leaves	NMR; IR; MS; UV	[58,61,62]
Stigmasterol	C_29_H_48_O	Dried leaves (Hexane extract)	GCMS	[62]
Stigmast-4-ene-3-one	C_29_H_48_O	Aerial parts	Spectroscopy	[61]
**Glycosides**				
Acteoside	C_29_H_36_O_15_	Arial parts (Methanol extract)	TLC; HPLC	[57]
Agastachoside	C_24_H_24_O_11_	Stem (Ethanol extract)	IR; HR; NMR	[60]
Apigenin-7-*O*-(3ʺ,6ʺ-di-(*E*)-*p*-coumaroyl)-β-d-galacto-pyranoside	C_30_H_26_O_12_	Leaf/Stem (Ethanol extract)	IR; HR; NMR	[58,60]
3α-Hydroxypatchoulol 3-*O*-β-d-glucopyranoside	C_21_H_36_O_7_	Air dried whole plant (Ethanol extract)	NMR	[63]
15-Hydroxypatchoulol 15-*O*-β-d-glucopyranoside	C_21_H_36_O_7_	Air dried whole plant (Ethanol extract)	NMR	[63]
Isocrenatoside	C_29_H_34_O_15_	Arial parts/Stem (Methanol/Ethanol extract)	TLC;HPLC; IR;HR;NMR	[58,59,60]
3ʺ-*O*-Methylcrenatoside	C_29_H_36_O_15_	Stem (Ethanol extract)	IR; HR; NMR	[58,59,60]
Soya-cerebroside I and II	C_40_H_75_NO_9_	Stem (Ethanol extract)	IR; HR; NMR	[60]
Tilianin	C_22_H_22_O_10_	Stem (Ethanol extract)	IR; HR-ESI-MS; NMR	[60]
Rubusoside	C_32_H_50_O_13_	Air dried whole plant (Ethanol extract)	NMR	[63]
**Triterpenes**				
Epifriedelinol	C_30_H_52_O	Leaves	Spectroscopy	[61]
Methyl oleanolate	C_31_H_50_O_3_	Aerial parts	Spectroscopy	[61]
**Sesquiterpenes**				
8α,9α-Dihydroxypatchoulol	C_15_H_26_O_3_	Arial parts (Methanol extract)	IR;NMR	[64]
3α,8α-Dihydroxypatchoulol	C_15_H_26_O_3_	Arial parts (Methanol extract)	IR;NMR	[64]
2β,12-Dihydroxypathoulol	C_15_H_26_O_3_	Arial parts (Methanol extract)	IR;NMR	[64]
10 α-Hydroperoxyguaia-1,11-diene	C_15_H_25_O_2_	Dried whole herb (Acetone extract)	IR; NMR	[65]
1 α-Hydroperoxyguaia-10(15),11-diene	C_15_H_25_O_2_	Dried whole herb (Acetone extract)	IR; NMR	[65]
15 α-Hydroperoxyguaia-1(10),11-diene	C_15_H_24_O_2_	Dried whole herb (Acetone extract)	IR; NMR	[65]
2-Keto-4β-hydroxyguai-1, 11-diene	C_15_H_22_O_2_	Air dried stem (Ethanol extract)	UV; IR; NMR; MS	[66]
4-Hydroxy-10-epi-rotundone	C_15_H_22_O_2_	Air dried stem (Ethanol extract)	UV; IR; NMR; MS	[66]
10 α-Hydroperoxyguaia-1,11-diene	C_15_H_25_O_2_	Dried whole herb (Acetone extract)	IR; NMR	[65]
1 α-Hydroperoxyguaia-10(15),11-diene	C_15_H_25_O_2_	Dried whole herb (Acetone extract)	IR; NMR	[65]
15 α-Hydroperoxyguaia-1(10),11-diene	C_15_H_24_O_2_	Dried whole herb (Acetone extract)	IR; NMR	[65]
6-Hydroxypatchoulol	C_15_H_26_O_2_	Arial parts (Methanol extract)	IR; NMR	[64]
Patchouli alcohol	C_15_H_26_O	Leaf (n-hexane extract)	TLC; HPLC; HRMS; EIMS	[61,62]
**Organic Acids**				
Dibutyl phthalate	C_16_H_22_O_4_	Aerial parts	Spectroscopy	[61]
Succinic acid	C_4_H_6_O_4_	Aerial parts	Spectroscopy	[58,59]
**Others**				
Tschimganical A	C_11_H_16_O_3_	Air dried stem (Ethanol extract)	UV; IR; NMR; MS	[62]
Uracil	C_4_H_4_N_2_O_2_	Stem (Ethanol extract)	IR; HR; NMR	[60]

Three new sesquiterpene hydroperoxides such as 10α-hydroperoxyguaia-1,11-diene, 1α-hydroperoxyguaia-10(15),11-diene and 15α-hydroperoxyguaia-1(10),11-diene were determined using chromatographic separation of an acetone extract of dried patchouli herb [65]. Similarly, Kongkathip *et al.* [62] obtained 0.05% dry weight of patchoulol, 0.09% dry weight of phytosterols such as β-sitosterol and stigmasterol and 0.04% dry weight of a flavonoid, 7,3ʹ,4-tri-*O*-methyl-eriodictyol. Wang *et al.* [60] identified 12 non-volatile compounds, including flavonoids and glycosides from the ethanol extract of *P. cablin* stems. The identified compounds were tilianin, diosmetin-7-*O*-β-d-glucopyranoside, 3ʺ-*O*-methylcrenatoside, uracil, soya-cerebroside I and II, agastachoside, apigenin-7-*O*-(3ʺ,6ʺ-di-(*E*)-*p*-coumaroyl)-β-d-galactopyranoside, 5-hydroxy-3,3ʹ,4ʹ,7-tetramethoxyflavone, 4ʹ,5-dihydroxy-3,3ʹ,7-trimethoxyflavone, acacetin, crenatoside and isocrenatoside. The compounds tilianin, diosmetin-7-*O*-β-d-glucopyranoside, uracil, soya-cerebroside I and II, agastachoside, apigenin-7-*O*-(3ʺ,6ʺ-di-(*E*)-*p*-coumaroyl)-β-d-galactopyranoside and acacetin were isolated for the first time. From ethanolic extracts of complete patchouli plants, the compounds 3α-hydroxypatchoulol 3-*O*-β-d-glucopyranoside, rubusoside (diterpene glycoside) and 15-hydroxy-patchoulol 15-*O*-β-d-glucopyranoside (sesquiterpene glycosides) were isolated for the first time [63]. Using 1D- and 2D-NMR techniques, Zhou *et al.* [64] isolated and elucidated the structures of four new patchoulol derivatives, namely 8α,9α-dihydroxypatchoulol, 3α,8α-dihydroxypatchoulol, 6-hydroxypatchoulol, and 2β,12-dihydroxypathoulol from the aerial parts of *P. cablin*. Four new sesquiterpenes, namely 8-keto-9(10)-α-patchoulene-4α-ol, 2-keto-1(5)-β-patchoulene-4β-ol, 2-keto-1(5)-β-patchoulene-4α-ol and 2-keto-4β-hydroxyguai-1,11-diene were isolated from the air dried powdered stems of *P. cablin* [66]. 

Zhou *et al.* [61] investigated the chemical constituents of the aerial parts of *P. cablin* and isolated 13 compounds using column chromatography and then used spectroscopic data to identify the following compounds; patchouliol, pogostone, friedelin, epifriedelinol, oleanolic acid, methyl oleanolate, 5α-stigmast-3,6-dione, stigmast-4-ene-3-one, β-sitosterol, pachypodol, retusin, (−)-guaiacylglycerol and dibutyl phthalate. The compounds, methyl oleanolate, 5α-stigmast-3,6-dione, stigmast-4-ene-3-one, (−)-guaiacylglycerol and dibutyl phthalate were isolated for the first time in this genus. A new HPLC-DAD method was developed to quantify both volatile and non-volatile components from patchouli [51]. Using this method pogostone (volatile) and eight flavonoids (non-volatile) such as apigenin, rhamnetin, ombuine, 4ʹ,5-dihydroxy-3,3ʹ,7-trimethoxyflavone, 5-hydroxy-7,3ʹ,4ʹ-trimethoxyflavanone, 5-hydroxy-3,3ʹ,4ʹ,7-tetramethoxyflavone, 3,5-dihydroxy-7,4ʹ-dimethoxy-flavone and 5-hydroxy-3,4ʹ,7-trimethoxyflavone can be determined simultaneously.

## 4. Pharmacological Activities

### 4.1. Antimicrobial Activities

#### 4.1.1. Antibacterial Activities

In traditional medicine, patchouli plants are used for treating common cold and fungal infections [67]. Among 10 essential oils studied for antibacterial and antifungal activity, patchouli oil was found to be more effective in inhibiting 20 bacterial strains and all 12 fungi [68]. The essential oils of *P. cablin* from three different geographic regions (China, India and Indonesia) were assessed *in vitro* against 17 pathogenic fungi and 16 commensal bacteria (from the skin, mucous membrane, nail, foot and armpit). The results revealed a clear antifungal and antibacterial activity of patchouli oil [69]. The essential oil of patchouli was effective in inhibiting *Acenitobacter baumanii*, *Aeromonas veronii*, *Candida albicans*, *Enterococcus faecalis*, *Escherichia coli*, *Klebsiella pneumonia*, *Pseudomonas aeruginosa*, *Salmonella enteric* and *Staphyllococcus aureus* [70]. Patchouli oil, tea tree oil, geranium oil, lavender oils and Citricidal^TM^ (seed extract of grapefruit) were used against bacteria. They also used patchouli oil for treating epidemic methicillin-resistant *S. aureus* infection and when compared to a blend of β-sitosterol and stigmasterol and 7,3ʹ,4-tri-*O*-methyleriodictyol, patchoulol and hexane extract of dried patchouli leaves evidenced higher antibacterial activity against *Staphylococcus aureus* and *Bacillus subtilis* [60,71]. The antimicrobial activity of patchouli oil was studied by using molecular docking technology and *in vitro* antimicrobial assay [72]. The study confirmed the strong antimicrobial effects. Mainly the constituents, pogostone and (−)-patchoulol had broader therapeutic prospects in bacterial infection. The aqueous and organic extracts of patchouli and geranium leaves showed significant antimicrobial properties against *E. coli*, *B. subtilis*, *S. aureus and E. aerogenes* [73]. Patchouli oil was effective in preventing biofilm formation by food borne pathogens, *Staphylococcus aureus* [74]. In recent times, green synthesis of nanoparticles was performed by using the essential oils of vanilla, patchouli and ylang-ylang. These nanobiosystems were very effective against adherence and biofilm formation by clinical strains of *Staphylococcus aureus* and *Klebsiella pneumonia* [75]. Patchouli oil also exhibited antimicrobial activity against the isolated enteric bacteria from common house lizard faecal droppings [76]. Selective antibacterial activity against *Helicobacter pylori* was exhibited by patchoulol without affecting the normal flora of the gastrointestinal tract. Patchoulol also possessed urease inhibitory potential and thus it can be used as a promising biomolecule to cure *H.  pylori* infections [77]. The gram-positive bacteria such as *Staphylococcus*, *Bacillus*, and *Streptococcus* species were successfully inhibited by using patchouli oil [78]. 

#### 4.1.2. Antifungal Activities

The medium having 100 µg/mL of an oil mixture (lemongrass, thyme, patchouli and cedarwood oils inhibited mycelial growth of *C. albicans* [79]. Patchouli alcohol (44.52%) of the plant essential oil showed antifungal activity against a population of *Aspergillus* species [80]. The potential of pogostone, a natural product from *P. cablin* effective in treatment of *Candida* infections, particularly for vulvovaginal candidiasis, was evidenced by Li *et al.* [34]. Similarly, patchouli oil effectively inhibited *C. albicans* [81]. Further, pogostone and its synthesized analogues showed effective activities against Gram-positive, Gram-negative bacteria and *C. albicans*. Based on molecular docking studies, it was suggestd that a promising antifungal agent can be obtained by appropriate structural modifications of pogostone analogues [33]. 

#### 4.1.3. Antiviral Activities

Traditional medicines from herbs, including patchouli were screened for ant-influenza viral activity. It was shown that about 10 μg/mL concentration of methanol extract of patchouli leaves could inhibit influenza virus A/PR/8/34 (H1N1) up to 99.8% and the IC_50_ values was estimated to be 2.6 μM [82]. Similarly, the results presented by Wu *et al.* [83] suggested anti-influenza A (H2N2) viral activities of patchoulol with an IC_50_ of 4.0 μM and thus making it a potent anti-influenza viral agent to be used by pharma industries. The *in vivo* anti-influenza virus effect of patchouli alcohol was studied using mouse as a model and the study confirmed that oral administration of patchouli alcohol (20 mg/kg to 80 mg/kg) augmented protection against influenza virus infection by improving the immune response and attenuating systemic as well as pulmonary inflammatory responses [84]. More recently, Wu *et al.* [85] observed anti-influenza viral properties using patchoulol (10 μg/mL) under *in vitro* condition and found that patchoulol enhanced the ability of innate immune recognition and response, and restrained the expression of IFN-α inflammatory factor to attenuate the inflammatory responses.

#### 4.1.4. Treatment of HIV/AIDS and Opportunistic Infections

In French hospitals, essential oils were used as suitable antimicrobial agents to treat specific opportunistic infections caused by *Candida albicans*, *Cryptococcus neoformans*, methicillin-resistant *Staphylococcus aureus* and *Herpes simplex* type I and II found in Acquired Immunodeficiency Syndrome patients [86].

### 4.2. Gastrointestinal Protective Activity

Water extract of *P. cablin* was shown to protect and maintain the membrane fluidity of intestinal epithelial cells by regulating the levels of nitric oxide and tumor necrosis factor in serum. This study provides an experimental basis for gastrointestinal protection against trauma or surgical operation [87]. 

### 4.3. Antiemetic Activity

Hexane extracts of patchouli plant leaves showed anti-emetic activity in young chicks. Patchoulol, pachypodol, pogostol, retusin and stigmast-4-en-3-one demonstrated anti-emetic properties at doses of 50–70, 10–50, 20–50 and 50 mg/kg, respectively [88]. 

### 4.4. Defaecation and Constipation

Mikuriya *et al.* [89] carried out an experiment to study the influence of patchouli oil on defecation and constipation using two mouse models; one having flaccid constipation and other with constipation because of lower fibrous food intake. It was found that in both models the number of faeces and its dry weight increased after smelling the patchouli oil aroma. Also, it was proved that olfactory neurotransmission systems responsible for overcoming constipation in mice.

### 4.5. Blood Coagulation and Fibrinolytic Activities

Blood coagulation and fibrinolytic activity of various essential oils including patchouli was studied using *in vitro* enzymatic reactions such as fibrin formation from fibrinogen by thrombin and fibrin resolution by urokinase. The study revealed that both coagulation and fibrinolytic activities were demonstrated by chamomile, eucalyptus and neroli oils while an efficient hyperfibrinolysis was revealed by the oils of citrus, pine, patchouli and frankincense [90]. 

### 4.6. Antithrombotic Activities

Park *et al.* [91] have examined in vitro, ex vitro and in vivo use of the herbal medicine Sunghyangjunggisan and its ingredients (P. cablin, Perilla frutescens, Arisaema amurensa, Aucklandia lappa, Atractylodes macrocephala, Citrus unshiu, and Ziziphus jujuba) as a novel antithrombotic agent. In vitro adenosine 5ʹ-diphosphate (ADP) and collagen induced rat platelet aggregations were not inhibited by Sunghyangjunggisan. However, Sunghyangjunggisan significantly inhibited ex vitro rat platelet aggregation. It also showed significant protection from death due to pulmonary thrombosis in mice.

### 4.7. Antioxidant Activities 

Patchouli oil showed an efficient free radical scavenging activity and inhibited the oxidation of hexanal to hexanoic acid [92]. Reactive oxygen species induced brain cell injury can be treated by using patchouli herb [93]. *P. cablin* protected from cell death due to necrosis and apoptosis induced by hydrogen peroxide in human neuroglioma cell line A172, thus suggesting its use for treating many neurodegenerative disorders*.* Patchouli oil prevented photoaging by exhibiting antioxidative property and maintained skin structural integrity caused by UV irradiation [94]. Similarly, the use of patchoulol increased the revitalization of UV-induced skin lesions through antioxidant and anti-inflammatory actions together with down-regulating the expression of matrix metalloproteinases (MMP-1 and MMP-3) [95]. Lipopolysaccharides present on the outer membrane of Gram-negative bacteria are responsible for causing mastitis and the study conducted by Li *et al.* [96] showed that patchouli alcohol was efficient in inhibiting TNF-α, IL-6, and IL-1β productions. It was also observed that patchouli alcohol attenuated mammary histopathologic changes, therefore patchouli alcohol can be used as a potent therapeutic reagent for preventing mastitis.

### 4.8. Analgesic and Anti-Inflammatory Activities

The methanol extract of patchouli plants was demonstrated to have analgesic and anti-inflammatory activity in mice. This validates the good acceptance of the patchouli herb in traditional medicinal practices [97]. Results of an analgesic study indicated that acetic acid induced writhing responses were decreased by using methanol extracts of patchouli (1.0 g/kg). The extracts of *Chrysanthemum indicum*, *P. cablin* and *Curcuma wenyujin* herbs, the components of the traditional medication recipe CPZ used in China, exhibited a strong anti-inflammatory response by regulating interleukin-1β (IL-1β) and prostaglandin E(2). Likewise, patchoulol effectively regulated the expression of TNF-α, IL-1β, IL-6, iNOS and COX-2 mRNAs in RAW264.7 cells due lipopolysaccharide induced inflammation [98]. Similarly, patchouli oil and ethanol extract of its root and rhizome exhibited strong *in vivo* anti-inflammatory properties [99,100]. The mechanism involved in anti-inflammatory property of patchoulol was investigated in mouse macrophage and human colorectal cancer cells [101]. More recently, Li *et al.* [35] revealed that pogostone possessed anti-inflammatory effect and suggested its use for development of a pharmaceutical drug to treat septic shock. Water extracts of patchouli herb suppressed colon inflammation through suppressing the expression of pro-inflammatory cytokines [102].

### 4.9. Antimutagenic Activity

In *Salmonella typhimurium* TA1535/pSK1002, *umu* gene expression due to SOS response stimulated due to mutagenic agent, 2-(2-furyl)-3-(5-nitro-2-furyl)acrylamide was suppressed by patchouli plant methanolic extract [103].

### 4.10. Effects on Tumors/Cancer Cells and the Immune System

The compounds 5,7-dihydroxy-3ʹ,4ʹ-dimethoxyflavanone, ombuin and licochalcone A were shown to exhibit cytotoxicity. The compound, licochalcone A showed PI- PLC gamma 1 inhibition activity. When licochalcone A was applied to promyelocyti leukaemia cells (HL-60), terminal differentiation along with the production of monocytes was observed [56]. However, patchoulol inhibited HeLa cell proliferation, suppressed cell differentiation and enhanced apoptosis in human colorectal cancer cell lines, HCT116 and SW480 [104,105]. According to these authors, patchouli alcohol inhibited histone deacetylase 2 expressions and histone deacetylase enzyme activity, down regulating c-myc and activating NF-κB pathway. A patchouli sesquiterpenoid, α-bulnesene was effective in inhibiting platelet-activating factor [106]. Anti-platelet activity was due to induction of intracellular signal transduction by α-bulnesene which interferes with activities of cyclooxygenase resulting in decreased thromboxane production. Now α-bulnesene is considered as a potent anti-platelet aggregation agent. The investigation on immunomodulatory potential of patchouli alcohol in Kumming mice revealed that patchouli alcohol activates the mononuclear phagocytic system and improves humoral immune response by suppressing the cellular immune response [107]. Xinxiang granule (XXG) is prepared from Biond magnolia flowers (*Magnolia biondii*), patchouli herb and small centipeda herb (*Centipeda minima*) and is used to treat allergic rhinitis and passive skin irritability [108]. Recently, the potential uses of patchouli oil for its antinociceptive and anti-allergic activities were confirmed in mouse models [109].

### 4.11. Effects against Skin Diseases 

The essential oils, including patchouli oil at 12% concentration, effectively controlled skin infections and odor in patients suffering ulcers, torn skin, skin abrasions and pressure sores. The healing duration was considerably reduced by the use of essential oils [110]. The structural integrity of UV irradiated skin was maintained by the application of patchouli essential oil and prevented photoaging and increased revival of skin lesions [97,98].

### 4.12. Pharmacokintetic Activities 

A simple and sensitive liquid chromatography tandem mass spectroscopy (LC-MS) method was developed to analyze pogostone in the plasma of rat after intravenous and oral administration [111]. This method is useful in pharmacokinetic studies of preclinical investigations. Lately, Li *et al.* [112] investigated the metabolic profile of pogostone *in vitro* and *in vivo* using LC-MS. Patchouli oil exhibited significant gastroprotective effects against gastric ulceration. According to them, the possible mechanism of anti-ulcerogenic potential of patchouli oil might be due to stimulation of COX-mediated PGE_2_, improvement of antioxidant and anti-inflammatory status, preservation of GBF and NP-SH, as well as boost of gastric mucus production [113].

### 4.13. Insecticidal Activity

*Cymbopogon nardus* and *P. cablin* leaf ashes at 1% (w/w) each caused pest mortality and were found to be effective repellents against *Stegobium paniceum* with 78% and a 64% repellency, respectively [114]. High fumigation insecticidal activity against four museum insect pests (*Lasioderma serricorne*, *Sitophilus zeamais*, *Tribolium confusum* and *Falsogastrallus sauteri*) by geranium oil, spikenard oil, muskmelon oil and patchouli oil was reported by Chun *et al.* [115]. Both patchouli oil and its main constituent, patchoulol showed toxicity and repellency towards Formosan subterranean termites (*Coptotermes formosanus* Shiraki). Topical application of patchoulol to the dorsum portion showed unusual tissue damage inside the exoskeleton of the termite [116]. Similarly, insecticidal activity against *Trypanosoma cruzi* was exhibited by the sesquiterpene hydroperoxides such as 10 α-hydroperoxyguaia-1,11-diene, 1α-hydroperoxyguaia-10(15),11-diene and 15α-hydro-peroxyguaia-1(10),11-diene [65]. Thirty eight essential oils were screened against the mosquito under laboratory conditions using human subjects. From the report it was noticed that the use of undiluted Patchouli oil was effective in providing two hours of complete mosquito repellency [117]. 

An eco-friendly, biodegradable insecticidal agent, consisting of petroleum ether extracts of *C. cassia*, *P. cablin* and *E. caryophyllata* can be used to control the house dust mite (*Dermatophagoides farina*) [118]. Patchouli oil showed strong repellency to three species of urban ants and possessed strong bio-insecticidal activity and particularly, patchouli alcohol had the most effective repellent activity against *Ades aegypti*, *Anopheles stephensi* and *Culex quinquefasciatus* mosquito bites [119,120]. Likewise, Phal *et al.* [121] evaluated herbal mosquito coils against *Aedes aegypti* mosquito and found a combination of patchouli and valamus (75:25) exhibiting significant knockdown activity at 7.5% concentration with all the coils (rice husk, corn cob and sawdust-based coils). In recent times, strong larvicidal activity, antifeedant activity, pupicidal activity and growth inhibition property were exhibited by progostone against *Spodoptera litura* and *Spodoptera exigua* thus making it a promising candidate to control various insects which destroy agricultural plants [122].

### 4.14. Aromatherapy

In aromatherapy, patchouli oil is used to help reduce tension, insomnia and anxiety. It’s wine-like intoxicating aroma functions as an aphrodisiac and helps to sharpen intelligence, improve concentration, and provide insight. Spiritually, it is used in incense sticks as it helps to create a calming atmosphere. In normal adult subjects, the fragrance inhalation effects on sympathetic activities was studied by measuring the amount of catecholamine content in the serum and monitoring fluctuations in blood pressure. There was a decreased sympathetic activity after inhalation of fragrance in normal subjects [123]. Aqueous cream containing lavender oil, sweet marjoram oil, patchouli oil and vetiver oil was applied five times a day onto the bodies and limbs of care facility residents and the results indicated a decreased frequency of dementia-associated behavior. Mental alertness and awareness in participants was improved by the use oils. The problems related to stress due to exhaustion and anxiety can be addressed by using essential oils as they stimulate adrenal hormone secretion [124]. The anti-inflammatory and cooling oils such as *P. cablin* and *Citrus limonum* were used for treating symptoms of menopause such as hot flashes and sweating. An aromatherapy oil blend of patchouli, with jasmine, ylang-ylang, sandalwood, rose and vetiver can definitely inspire clarity and a harmonious flow of energy that interacts with thyroid glands and balances hormonal secretion [125]. Various psychiatric disorders can be treated by aromatherapy. Patchouli oil influences cerebral functions including calming, sedative and uplifting [126]. The vaporized mixtures of essential oils (patchouli, orange, ylang ylang, rosemary, basil, peppermint, geranium, bergamot, rosewood, chamomile and jasmine) reduced disturbed behavior among 10 patients with dementia [127]. The physiological responses such as blood pressure, pulse rate, stress index and brain waves were reduced after sniffing patchouli oil odor. This suggests that patchouli oil odor improves mood and possess curative effects when its fragrance is inhaled by humans. The influence of odor was due to its main compound, patchouli alcohol [128]. Aromatherapy (inhalation, compresses, baths and massages) can help to manage stress and reduce perceptions of chronic pain [86].

## 5. Conclusions and Recommendations

*P. cablin* is being explored globally to obtain key chemical compounds for making new drug molecules with great therapeutic potential as well as in the fragrance industry. This review summarizes the updated research studies on the phytochemisty and pharmacological effects of *P. cablin.* The present information helps in bridging the gap between modern scientific studies and available traditional medical reports on this plant. As chemical constituents of *P. cablin* vary depending on the origin and plant parts, a protocol needs to be standardized to isolate and obtain pure compounds. Though various chemical compounds have been isolated, purified and characterized, many compounds are yet to be studied in detail. Till now, limited efforts are made in the pharmacokinetics investigations related to the mechanism of action of the individual isolated compounds under *in vivo* conditions. Also, research should be emphasized on the toxicity and safety aspects of *P. cablin* compounds using animal models. Further, therapeutic prospects of many new chemical compounds from *P. cablin* under *in vitro* and *in vivo* conditions should be explored in detail.
